# Long noncoding RNA NEAT1 promotes cell proliferation, migration, and invasion in hepatocellular carcinoma through interacting with miR‐384

**DOI:** 10.1002/jcb.27499

**Published:** 2018-10-22

**Authors:** Liying Zhu, Nenghong Yang, Chengcheng Li, Guoqi Liu, Wei Pan, Xing Li

**Affiliations:** ^1^ Department of Medical Laboratory Affiliated Hospital of Guizhou Medical University Guiyang Guizhou China; ^2^ Department of Medical Laboratory Guizhou Medical University Guiyang Guizhou China; ^3^ Department of Hepatobiliary Surgery Affiliated Hospital of Guizhou Medical University China; ^4^ Prenatal Diagnosis Center, Affiliated Hospital of Guizhou Medical University and Guizhou Provincial Prenatal Diagnosis Center

**Keywords:** cell proliferation, hepatocellular carcinoma (HCC), migration, MiR‐384, nuclear enriched abundant transcript 1 (NEAT1), invasion

## Abstract

It was reported that long non‐coding RNA nuclear‐enriched abundant transcript 1 (NEAT1) is involved in hepatocellular carcinoma (HCC). However, the underlying mechanism of tumorigenesis is still largely unclear. Here, we found that NEAT1 is remarkably upregulated in HCC tissues and cell lines. Overexpression of NEAT1 notably accelerated HCC cell proliferation, migration, and invasion. Knockdown of NEAT1 significantly inhibited HCC cell proliferation, migration and invasion. MiR‐384 expression was lower in HCC tissues and cell lines than adjacent nontumor tissues and L02 cell. MiR‐384 exhibited the functions of tumor‐suppressive. The expression of miR‐384 was negatively correlated with the expression of NEAT1. Overexpression of NEAT1 reduced miR‐384 expression, whereas inhibition of miR‐384 led to a distinct upregulation of NEAT1 expression. In addition, we provided evidence that miR‐384 was directly bound to the sequence of NEAT1 by luciferase reporter and RNA‐binding protein immunoprecipitation assays. Overexpression of miR‐384 inhibited NEAT1 function. Thus, we demonstrated that NEAT1 promotes the malignant biological properties of hepatocellular carcinoma by negatively regulating miR‐384.

## 1 INTRODUCTION

Hepatocellular carcinoma (HCC) is one of most common cancers worldwide and lists as the third leading cause of cancer‐related deaths around the world. The process of HCC involves complicated steps. Thus, studying the pathogenesis of HCC is particularly important.

Long non‐coding RNAs (lncRNAs) are non–protein coding transcripts and longer than 200 nt.^1^, ^2^ Recently, emerging reports have shown that numerous lncRNAs and microRNAs (miRNAs) are involved in multiple pathological steps of HCC, including cell proliferation, angiogenesis, cellular signaling, and metastasis.^3^, ^4^ The LncRNA CRNDE promotes hepatic carcinoma cell proliferation, migration, and invasion by suppressing miR‐384.^5^ The LncRNA MEG3 inhibits cellular proliferation via negative modulation of miRNA‐664 in hepatocellular carcinoma.^6^ MALAT1, H19, HULC, and HOTAIR have been found to act as oncogenes or tumor suppressors.^7^, ^8^, ^9^

## 2 MATERIALS AND METHODS

### 2.1 HCC tissue samples and cells culture

Twelve pairs of tumor and adjacent nontumor tissues were obtained from surgical resections of HCC in the affiliated hospital of Guizhou medical university. The specimens were snap‐frozen in liquid nitrogen and stored at −80°C. All of the patients received surgery, without preoperative chemotherapy or radiation therapy. All patients provided a written informed consent, and ethical approval was granted from the Committee for Ethical Review of Research involving the affiliated hospital of Guizhou Medical University (Guizhou, China).

HCC cell lines (SMMC7721, SK‐hep1, Huh7, HepG2), human immortalized, normal human liver cell line (L02), and the cell line 293T were stored in our lab. They were cultured in Dulbecco modified Eagle medium of high glucose with 10% fetal bovine serum (FBS, BI, ISR). All of the cells were incubated at 37°C with 5% CO_2_.

### 2.2 RNA, miRNA isolation, and quantification real‐time polymerase chain reaction

Total RNA was isolated from the tissues and the cell lines with trizol reagent (Life Technologies Corporation, Carlsbad, CA). RNA was then reverse transcribed to cDNA with an M‐MLV reverse transcriptase kit (TAKALA, Dalian, China). SYBR‐Green real‐time PCR was used to perform quantification real‐time polymerase chain reaction (qRT‐PCR) assay (TAKALA, Dalian, China). cDNA from miRNAs was then generated with a miRNA cDNA Synthesis Kit (CWBIO, Beijing, China). A 2× miRNA qPCR Mixture Kit were used to perform qRT‐PCR assay (CWBIO, Beijing, China). The expression of RNA or miRNA was normalized to glyceraldehyde 3‐phosphate dehydrogenase (GAPDH) or U6 and calculated using the 2^−ΔΔ*C*t^ method. The primers used in this study are as follows: NEAT1: forward 5′‐ATGCCACAACGCAGATTGAT‐3′, reverse 5′‐CGAGAAACGCACAAGAAGG‐3′; miR‐384: forward 5′‐GCGGCGGGCGATTCCTAGAAATTGTTCATA‐3′; GAPDH: forward 5′‐GGTCTCCTCTGACTTCAACA‐3′, reverse 5′‐GTGAGG GTCTCTCTCTTCCT‐3′; and U6: forward 5′‐GCTTCGGCAGCACATATACTAAAAT‐3′, reverse 5′‐CGCTTCACGAATTTGCGTGTCAT‐3′.

### 2.3 Plasmid construction and cell transfection

The full‐length sequences of human NEAT1 were cloned into pcDNA3.1 vector at the EcoRV/KpnIrestriction sites. The short‐hairpin RNA targeting human NEAT1 were ligated into the pGreenPuro shRNA vector (SBI, Palo Alto) according to the manufacturer's protocol. The sh‐RNA sequences of NEAT1: forward 5′‐GATCCGGGTTGGTTAGAGATACAGTGCTTCCTGTCAGACACTGTATCTCTAACCAACCCTTTTTG‐3′, reverse 5′‐AATTCAAAAAGGGTTGGTTAGAGATACAGTGTCTGACAGGAAGCACTGTATCTCTAACCAACCCG‐3′; sh‐NC: forward 5′‐GATCCGATGAAATGGGTAAGTACATTCAAGAGATGTACTTACCCATTTCATCTTTTTG‐3′, reverse 5′‐AATTCAAAAAGATGAAATGGGTAAGTACATCTCTTGAATGTACTTACCCATTTCATCG‐3′. A miR‐384 mimic, a miR‐384 inhibitor, and their negative controls were purchased from Invitrogen company (Gene Pharma, Shanghai, China). Cells were transfected with the Lipofectamine 3000 (Invitrogen, Carlsbad, CA) according to the manufacturer's instructions, and the effect of transfection was evaluated by qRT‐PCR.

### 2.4 Cell proliferation assay

Cell proliferation assays were performed with CellTiter 96® Aqueous One Solution Cell Proliferation Assay (MTS; Promega, Madison, WI). After the cells were transfected, 2000 cells were seeded into a 96‐well plate. When the cells were completely adherent to the plate, the time point was as 0 hour. At the same time, MTS solution (20 μL; Promega, Sunnyvale, CA) was added into each well and then incubated for 2 hours. The absorbance was determined at 490 nm with a microplate reader (Bio‐Tek Instruments). The absorbance was detected every 24 hours.

### 2.5 Colony‐formation assay

After the cells were transfected, 1000 cells were seeded into the six‐well plates and then cultured for 14 days. The cell colonies were visualized by a crystal violet cell colony staining kit, and the number of colonies was counted with ImageJ software.

### 2.6 Cell migration and invasion assay

Chambers with 8 μm pore size (Costar 3422; Corning, NY) were used in the study. The chambers were placed in 24‐well plates.The chambers were pre‐coated with 500 ng/mL Matrigel solution (BD, Franklin Lakes, NJ) for invasion assay. A total of 5 × 10^4^ cells with 100 μL serum‐free media were seeded into the upper chamber, and 600 μL 10% FBS medium was placed in the lower chamber. After incubation for 48 hours, the chambers were removed from the plates, and the cells on the top side of the chamber were wiped with a cotton swab. Migrating or invading cells were fixed with 4% paraformaldehyde and then stained by 1% crystal violet. The cells were counted with ImageJ software. Five randomly fields were counted, and photos were stored.

### 2.7 Western blot analysis

Total proteins were extracted from differently treated HCC cells with RIPA buffer (Beyotime, Shanghai, China) on ice. The proteins were isolated by sodium dodecyl sulfate–polyacrylamide gel electrophoresis and then transferred onto polyvinylidene fluoride membranes (Merck Millipore, Darmstadt, German). Next, the membranes were incubated with 5% non–fat milk in Tris‐buffered saline for 1.5 hours at room temperature and then incubated with primary antibodies as follows: CyclinD1 (diluted 1:500; Bioworld Technology, Nanjing, China), MMP2 (diluted 1:500; Bioworld Technology, Nanjing, China), and MMP9 (diluted1:400; Bioworld Technology, Nanjing, China). The secondary antibodies applied HRP‐conjugated goat anti‐rabbit IgG (diluted 1:2000; Bioworld Technology, Nanjing, China) for 2 hours at room temperature. GAPDH was used as a loading control. The blot was visualized by enhanced chemiluminescence (ECL kit; Advansta) and scanned with an ECLTM chemiluminescence detection system (Pierce).

### 2.8 Reporter vectors construction and luciferase assays

The NEAT1 full length was amplified by PCR and cloned into a PGL3‐control (Promega) to construct luciferase reporter vector (NEAT1‐Wt). The sequence of binding sites (miR‐384) was replaced with the mutated binding site of NEAT1 and was named NEAT1‐Mut vector. 293T and SMMC7721 cells were seeded into 96‐well plates, and the cells were co‐transfected with NEAT1 ‐Wt (or NEAT1‐Mut) and a miR‐384 mimic or scrambled NC when they reached 50%‐70% confluence. The luciferase activities were measured with the Dual‐Luciferase reporter assay kit (Promega) after transfection for 48 hours.

### 2.9 RNA‐binding protein immunoprecipitation

SMMC7721 cells were lysed with a complete RNA lysis buffer from an EZ‐Magna RNA‐binding protein immunoprecipitation (RIP) RNA‐binding protein immunoprecipitation kit (Millipore, Billerica, MA) according to the manufacturer's protocol. The cell lysate of the control groups and the miR‐384‐inhibitor (miR‐384‐in) groups were incubated with RIP immunoprecipitation buffer containing magnetic beads conjugated with human anti‐Argonaute2 (Ago2) antibody (Abcam, Cambridge, MA) and negative control normal mouse IgG (Proteintech, Wuhan, China). The samples were incubated with Proteinase K buffer, and then immunoprecipitated RNA was isolated. The RNA concentration was measured by a NanoDrop (Thermo Fisher Scientific). Furthermore, purified RNA was obtained and analyzed by qRT‐PCR to demonstrate the presence of the binding targets.

### 2.10 Statistical analysis

Statistical analyses were performed with SPSS 17.0 software (SPSS, Chicago, IL). The Student *t*‐test was used in the study. Each experiment was performed at least three times. A *P*‐value < 0.05 was considered to indicate statistical significance.

## 3 RESULTS

### 3.1 NEAT1 was significantly upregulated in HCC tissue and cells

**Figure 1:**
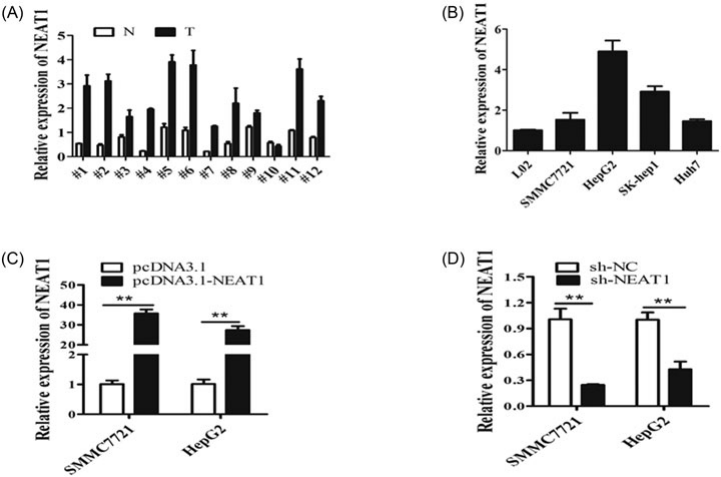
NEAT1 was significantly upregulated in HCC tissues and cell lines. A, NEAT1 expression level was examined in 12 paired HCC tissues (T) and nontumor tissues (N) by qRT‐PCR. Transcript levels were normalized to GAPDH expression. B, NEAT1 expression level was examined in HCC cell lines (SMMC7721, Huh7, SK‐hep1, and HepG2) and one normal liver cell line L02 by qRT‐PCR. Transcript levels were normalized to GAPDH expression. C, NEAT1 expression level was examined after the cells were transfected with pcDNA3.1, pcDNA3.1‐NEAT1, sh‐NC, sh‐NEAT1 at 48 hours in SMCC7721 and HepG2 cells. Transcript levels were normalized to GAPDH expression. **P < 0.01. GAPDH, glyceraldehyde 3‐phosphate dehydrogenase; HCC, hepatocellular carcinoma; NEAT1, nuclear‐enriched abundant transcript 1; qRT‐PCR, quantification real‐time polymerase chain reaction

The expression of NEAT1 in 12 paired HCC tissues (T) and nontumor tissues (N) was compared by qRT‐PCR. We found that the NEAT1expression was higher in the 12 paired HCC tissues than the nontumor tissues (Figure 1A). Furthermore, NEAT1 was obviously upregulated in HCC cell lines (SMMC7721, Huh7, SK‐hep1, and HepG2) when compared with that in L02 cell (Figure 1B).

SMMC7721 and HepG2 cells were selected for functional experiments. We first assessed the effect of overexpression of NEAT1 (pcDNA3.1‐ NEAT1) and knockdown of NEAT1 (sh‐ NEAT1). We found that NEAT1 drastically increases after transfection with the pcDNA3.1‐ NEAT1 plasmid (Figure 1C) and obviously decreases after transfection with the sh‐ NEAT1 plasmid for 48 hours (Figure 1D).

### 3.2 NEAT1 promotes cell proliferation, migration, and invasion of hepatocellular carcinoma

**Figure 2:**
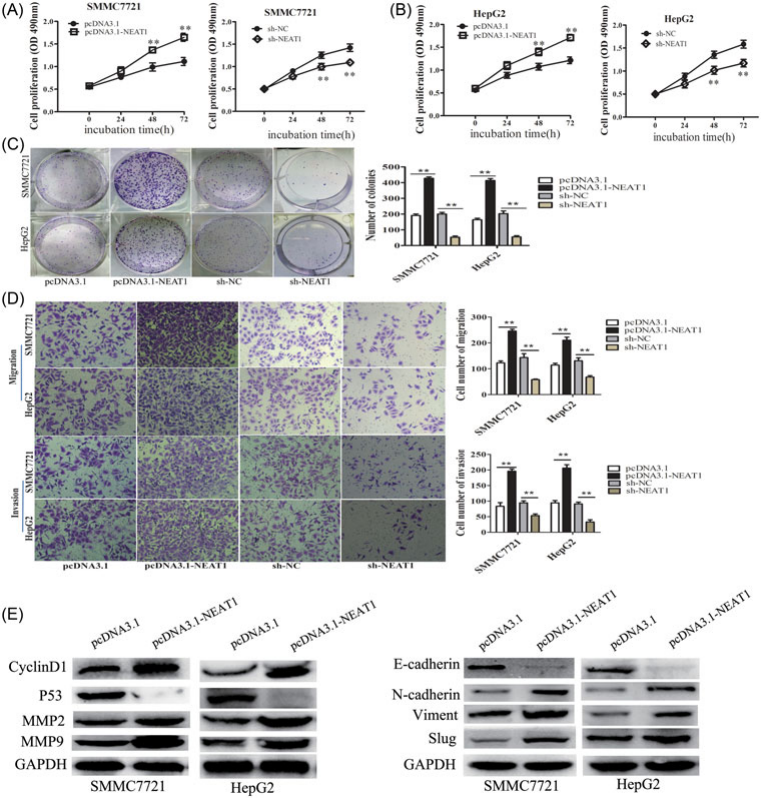
NEAT1 promoted cell proliferation, migration and invasion in HCC. (A,B) MTS cell proliferation was detected after cells were transfected with pcDNA3.1, pcDNA3.1‐NEAT1, sh‐NC, sh‐NEAT1 at 0, 24, 48 and 72 hour in SMCC7721 and HepG2 cells. Representative graphs are shown. (C) Cell colonies were calculated after cells were transfected with pcDNA3.1, pcDNA3.1‐NEAT1, sh‐NC, sh‐NEAT1 at 14 days in SMCC7721 and HepG2 cells. Representative graphs are shown. The data graphs depict the count number from three independent experiments. (D) Cell migration and invasion assay was examined and cell number was calculated after cells were transfected with pcDNA3.1, pcDNA3.1 ‐NEAT1, sh‐NC, sh‐NEAT1 at 24 hour in SMCC7721 and HepG2 cells. Data are shown as mean ± s.d. (n = 3) and are representative of three independent experiments. Scale bars = 100 μm. (E) The relative protein expression of CyclinD1, MMP2 and MMP9 was examined after cells were transfected with pcDNA3.1, pcDNA3.1‐NEAT1, sh‐NC, sh‐NEAT1 at 48 h in SMCC7721 and HepG2 cells. **P < 0.01

The MTS assay results verified that overexpression of NEAT1 significantly increased cell proliferation, and knockdown of NEAT1 dramatically inhibited the cell proliferation compared with the control group at 48 and 72 hours. (Figure 2A,B). Colony formation assay showed that overexpression of NEAT1 displayed more clones, yet the clone number was significantly decreased after knockdown of NEAT1 compared with control groups (Figure 2C). Transwell chamber assay confirmed the positive effect of NEAT1 on HCC cell migratory capacity, with decreased cell migration after NEAT1 knockdown in both SMMC7721 and HepG2 cell lines (Figure 2D). Furthermore, the effects of NEAT1 on cell invasion were assessed by transwell invasion assay (matrigel‐coated transwell); the cell invasive number was significantly increased after overexpression of NEAT1, whereas knockdown of NEAT1 showed opposite effects, which was exactly in line with the expectations (Figure 2D). Moreover, we also demonstrated that overexpression of NEAT1 upregulated the cell proliferation–related protein Cyclin D1, the cell migration and invasion–related protein MMP2 and MMP9 expression, whereas knockdown of NEAT1 downregulated Cyclin D1, MMP2, and MMP9 expression in SMMC7721 and HepG2 cell lines (Figure 2E,F). Taken together, these results suggested that NEAT1 promoted cell proliferation, migration, and invasion of HCC and exhibited an oncogenic property in HCC tumorigenesis.

### 3.3 MiR‐384 was downregulated in HCC and served as tumor suppressor

**Figure 3:**
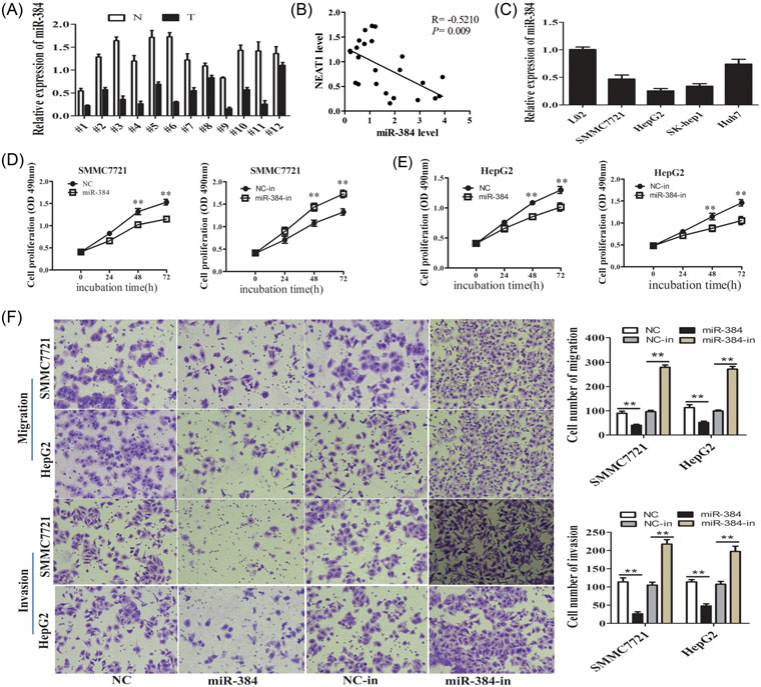
MiR‐384 was downregulated in HCC and functioned as a tumor suppressor. A, MiR‐384 expression level was examined in 12 paired HCC tissues (T) and nontumor tissues (N) by qRT‐PCR, using U6 as an internal control. B, The expression of NEAT1 was negatively correlated with that of miR‐384 (*r* = −0.5210, *P* = 0.009) in clinical specimens. C, MiR‐384 expression level was examined in HCC cell lines (SMMC7721, Huh7, SK‐hep1, and HepG2) and one normal liver cell line L02 by qRT‐PCR, using U6 as an internal control. D,E, MTS cell proliferation was detected after cells were transfected with NC, miR‐384mimic, NC‐in, miR‐384‐in at 0, 24, 48, and 72 hours in SMCC7721and HepG2 cells. Representative graphs are shown. F, Cell migration and invasion assay was examined and cell number was calculated after cells were transfected with NC, miR‐384mimic, NC‐in,miR‐384‐in at 24 hours. Representative graphs are shown. **P < 0.01. HCC, hepatocellular carcinoma; NEAT1, nuclear‐enriched abundant transcript; qRT‐PCR, quantification real‐time polymerase chain reaction

The QRT‐PCR results showed that miR‐384 is downregulated in the 12 paired HCC tissues compared with the nontumor tissues (Figure 3A), resulting in a significant inverse correlation between NEAT1 and miR‐ miR‐384 (Figure 3B). The expression of miR‐384 was lower in HCC cell lines than L02 cells (Figure 3C). The miR‐384 mimic (miR‐384) inhibited HCC cell proliferation by MTS assay, whereas promoted HCC cell proliferation after transfection with the miR‐384 inhibitor (miR‐384‐in; Figure 3D,E). Transwell chamber assay confirmed the negative effect of miR‐384 on HCC cell migratory capacity, with increased cell migration after transfection with the miR‐384 inhibitor (miR‐384‐in) in SMMC7721 and HepG2 cell lines (Figure 3F). In matrigel‐coated transwell assay, the cell invasive number was significantly decreased after transfection with the miR‐384 mimic, whereas inhibited miR‐384 (miR‐384‐in) showed the opposite effects (Figure 3F). In contrast to NEAT1, miR‐384 might act as a tumor suppressor and inhibit the proliferation, migration, and invasion of HCC cells.

### 3.4 MiR‐384 was a target of NEAT1 in HCC cells

**Figure 4:**
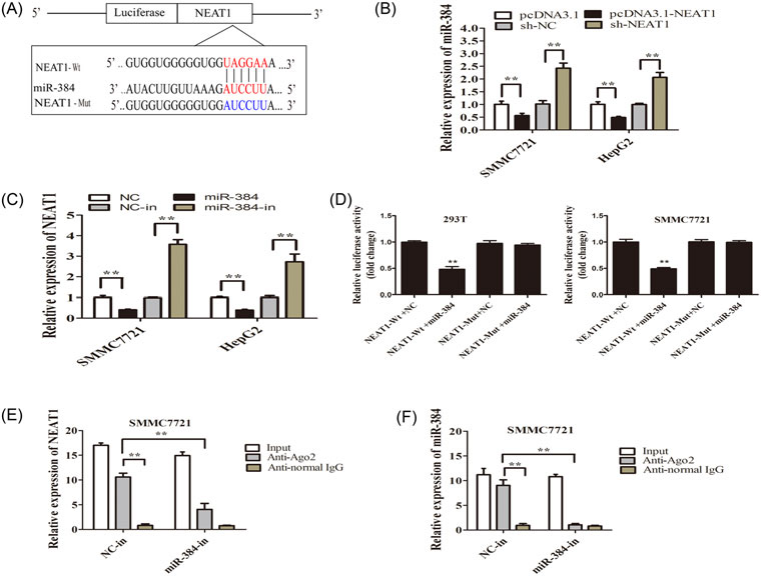
MiR‐384 was a target of NEAT1 in HCC cells. A, The relative miR‐384 expression level after being transfected with pcDNA3.1, pcDNA3.1‐NEAT1, sh‐NC, sh‐NEAT1 at 48 hours in SMCC7721 and HepG2 cells, using u6 as an internal control. B, The relative NEAT1 expression level after being transfected with NC, a miR‐384mimic, NC‐in, miR‐384‐in SMCC7721, and HepG2 cells, using GAPDH as an internal control. C, The NEAT1‐Wt luciferase reporter vector and a NEAT1‐Mut luciferase reporter vector by sequentially mutating the predicted miR‐384 sites in NEAT1 were constructed. D, MiR‐384 mimic reduced the luciferase activity of PGL3‐NEAT1‐Wt luciferase reporter vector, but not of PGL3‐NEAT1‐Mut luciferase reporter vector in 293T and SMMC7721 cells. E,F, The amount of NEAT1 and miR‐384 bound to Ago2 or IgG was measured by qRT‐PCR after transfection with a miR‐384 inhibitor or negative control in SMMC7721 cells. **P < 0.01. HCC, hepatocellular carcinoma; NEAT1, nuclear‐enriched abundant transcript; qRT‐PCR, quantification real‐time polymerase chain reaction

Accumulating studies showed that lncRNA might be a competing ceRNA for specific miRNAs to regulate its target genes.^10^ Using bioinformatics databases (Starbase v2.0 and miRanda), we found that miR‐384 had complementary sites with NEAT1 (Figure 4A). We confirmed that compared with adjacent normal tissues and L02 cells, miR‐384 expression was significantly lower in HCC tissues and cell lines (Figure 3A,B). To further demonstrate the relationship between NEAT1 and miR‐384, we firstly detected the expression of miR‐384 in pcDNA3.1‐NEAT1 and sh‐ NEAT1 cells by qRT‐PCR. We found that the expression of miR‐384 was downregulated in the pcDNA3.1‐NEAT1 group compared with the pcDNA3.1 group, while miR‐384 was upregulated in the sh‐NEAT1 group (Figure 4B). Then, the expression of NEAT1 was detected when the HCC cells were transfected with the miR‐384 mimic (miR‐384) or miR‐384 inhibitor (miR‐384‐in). We found that the expression of NEAT1 was downregulated in the miR‐384 mimic group compared with the control group (NC), while NEAT1 was upregulated in the miR‐384 inhibitor group compared with the inhibitor control group (NC‐in; Figure 4C).

To investigate the underlying mechanism of the lncRNA/miRNA regulatory function, dual‐luciferase reporter assays were conducted to determine the binding sites of NEAT1 and miR‐384. We constructed PGL3‐NEAT1 wild‐type (NEAT1‐Wt) and PGL3‐NEAT1 mutant‐type (NEAT1‐Mut) luciferase reporter plasmids. We transfected 293T and SMMC7721 cells using the PGL3‐wild‐type (Wt) NEAT or PGL3‐mutated (Mut) NEAT1 reporter vector and a miR‐384 mimic or NC. The luciferase activity in the NEAT1‐Wt+miR‐384 group was significantly attenuated than that in the control group, while the luciferase activity in the NEAT1‐Mut group was not affected (Figure 4D).

Previous studies have demonstrated that miRNAs are present in the form of miRNA ribonucleoprotein complexes (miRNPs) that contain Ago2, the key component of the RNA‐induced silencing complex (RISC).^11^, ^12^ We revealed that NEAT1 and miR‐384 were enriched in Ago2 immunoprecipitates compared with control IgG immunoprecipitates by RIP assay (Figure 4E,F). Taken together, these results indicated that both NEAT1 and miR‐384 are probably in the same RISC complex, and there might be a reciprocal repression feedback loop between NEAT1 and miR‐384.

### 3.5 Overexpression of miR‐384 largely inhibited NEAT1‐induced oncogenetic effects on HCC cells

**Figure 5:**
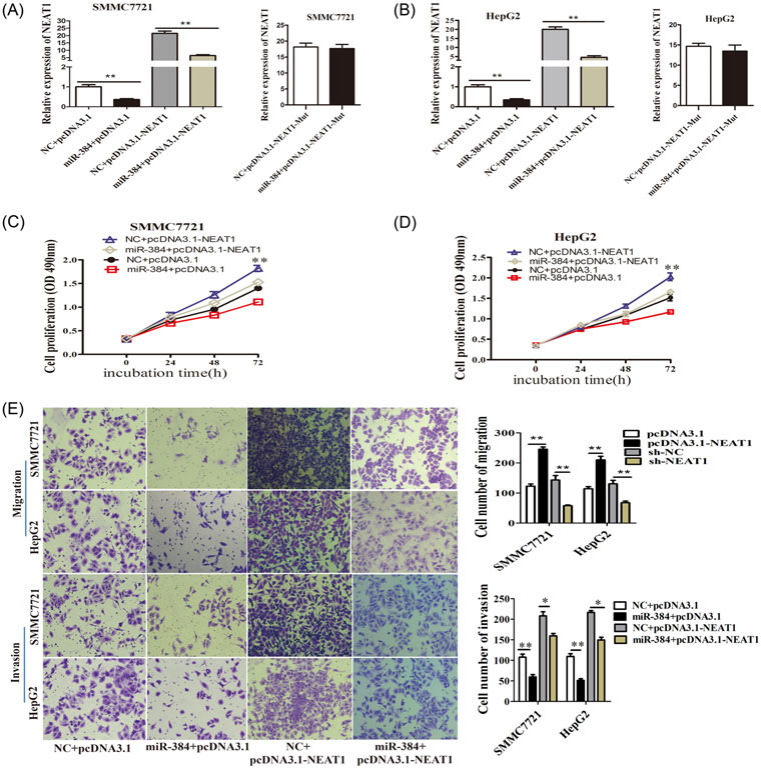
Overexpression of miR‐384 largely inhibited NEAT1‐induced oncogenetic effects on HCC cells. A,B, SMCC7721and HepG2 cells were cotransfected with a miR‐384 mimic and NEAT1 expression plasmid and the effect of miR‐384 on ectopically expressed NEAT1 was analyzed by qRT‐PCR, using GAPDH as an internal control. C,D, SMCC7721and HepG2 cells were cotransfected with negative control or miR‐384 mimic and pcDNA3.1 or pcDNA3.1‐NEAT1, cell proliferation was determined using MTS assays. Representative graphs are shown. E, Cell migration and invasion assays of HCC cells after cotransfection with negative control or miR‐384 mimic and and pcDNA3.1 or pcDNA3.1‐NEAT1. Representative graphs are shown. *P < 0.05, **P < 0.01. GAPDH, glyceraldehyde 3‐phosphate dehydrogenase; HCC, hepatocellular carcinoma; NEAT1, nuclear‐enriched abundant transcript; qRT‐PCR, quantification real‐time polymerase chain reaction

We further detect whether NEAT1 regulated cell proliferation, migration, and invasion by negatively regulating miR‐384 expression in HCC. MiR‐384 could significantly decrease the NEAT1 expression in both cell lines, but had nearly no effect on the expression of mutant NEAT1 (Figure 5A). MiR‐384 could inhibit the effect of NEAT1 on promoting cell proliferation upon co‐transfection with miR‐384 mimic and pcDNA3.1‐NEAT1 plasmids (Figure 5B). Moreover, the cell migration and invasion ability was inhibited after cotransfection of the miR‐384 mimic and pcDNA3.1‐NEAT1 plasmids (Figure 5C). Thus, these results indicated that NEAT1 promoted cell proliferation, migration, and invasion by sponging miR‐384 in HCC.

## 4 DISCUSSION

Numerous studies have shown that NEAT1, a lncRNA, is aberrantly expression in various cancers. The lncRNA NEAT1 enhances epithelial‐to‐mesenchymal transition and chemoresistance via the miR‐34a/c‐Met axis in renal cell carcinoma.^13^ Oct4 transcriptionally regulates the expression of lncRNAs NEAT1 and MALAT1 to promote lung cancer progression.^14^ LncRNA NEAT1 promotes proliferation and invasion via targeting miR‐181a‐5p in non‐small cell lung cancer.^15^ lncRNA NEAT1 overexpression is associated with unfavorable prognosis in patients with hepatocellular carcinoma after hepatectomy, accoring to a Chinese population‐based study.^16^ Long noncoding RNA NEAT1 promotes cell proliferation and invasion by regulating hnRNP A2 expression in hepatocellular carcinoma cells.^17^ In this study, our results revealed that NEAT1 was higher in HCC tissues compared with paired adjacent normal tissues. Biological function assays results showed that NEAT1 promoted cell proliferation, migration, and invasion in HCC. Knockdown of NEAT1 downregulated the cell proliferation related Cyclin D1 expression, migration, and invasion–related MMP2 or MMP9 expression in HCC cells.Thus, we demonstrated that NEAT1 acted as an oncogene in HCC, which was consistent with the above studies.

Accumulating evidence has shown that lncRNA might be a competing ceRNA for specific miRNAs to regulate its target genes.^10^ lncRNA NEAT1 has been reported to promote tumor progression by sponging some microRNAs. lncRNA NEAT1 regulates the permeability of the blood‐tumor barrier via miR‐181d‐5p‐mediated expression changes in ZO‐1, occludin, and claudin‐5.^18^ The lncRNA NEAT1 regulates epithelial to mesenchymal transition and radioresistance in through miR‐204/ZEB1 axis in nasopharyngeal carcinoma.^19^ The results of our study showed that miR‐384 was downregulated in HCC tissues and cell lines. MiR‐384 suppressed cell proliferation, migration, and invasion in HCC. First, overexpression of NEAT1 reduced miR‐384 expression, whereas inhibition of miR‐384 expression resulted in a significant upregulation of NEAT1, suggesting that NEAT1 was negatively regulated by miR‐384. Moreover, we provided evidence that miR‐384 targeted NEAT1 by directly binding to miRNA‐binding sites in NEAT1 sequence. In addition, the luciferase reporter assay revealed that miR‐384 was a target of NEAT1.

Studies have reported that small RNAs can have a broader range of function by binding to Argonaute proteins and associating with complementary RNA targets in the nucleus, including lncRNAs and pre‐mRNA.^20^ Previous studies have demonstrated that miRNAs are present in the form of miRNA ribonucleoprotein complexes (miRNPs) that contain Ago2, the key component of the RISC.^11^, ^12^ Studies have reported that Ago proteins can be imported into the nucleus 0,^21^, ^22^, ^23^ and miRNAs might be co‐imported with the Ago proteins into the nucleus.^24^, ^25^ Fluorescence correlation and cross‐correlation spectroscopy revealed Ago2 and Ago2‐small RNA complexes in the nucleus, and also suggested that Ago2‐small RNA complexes are predominantly loaded in the cytoplasm and imported in to the nucleus.^26^ In this study, RIP assay revealed that while NEAT1 was detected in Ago2 immunoprecipitates compared with the control group, its levels were drastically reduced in Ago2 complexes purified from cells treated with a miR‐384 inhibitor (Figure 4E,F), indicating that NEAT1 was likely present in the miR‐384‐RISC complex. In the study, overexpressed miR‐384 inhibited NEAT1 function.Therefore, the effect of NEAT1 on HCC cells' proliferation, migration, and invasion was through directly binding to the miR‐384.

In summary, we showed that the lncRNA NEAT1 was upregulated in HCC. Its effects on cell proliferation, migration, and invasion suggested that it exhibited oncogenic property in HCC tumorigenesis. MiR‐384 directly targeted NEAT1, suppressed NEAT1 expression and function, while NEAT1 acted as a molecular sponge for miR‐384. This reciprocal repression of miR‐384 and NEAT1 might highlight the significance of RNA‐RNA interaction and provide new insights into the mechanisms of tumorigenesis, including tumor growth, migration, and invasion.
